# Interleukin-32-induced thymic stromal lymphopoietin plays a critical role in macrophage differentiation through the activation of caspase-1 *in vitro*

**DOI:** 10.1186/ar4104

**Published:** 2012-11-28

**Authors:** Hyun-Ja Jeong, Sun-Young Nam, Hyun-A Oh, Na-Ra Han, Young-Sick Kim, Phil-Dong Moon, Seung-Youp Shin, Min-Ho Kim, Hyung-Min Kim

**Affiliations:** 1 Biochip Research Center, Hoseo University, 165, Sechul-ri, Baebang-myun, Asan, Chungnam 336-795, Republic of Korea; 2 Department of Pharmacology, College of Oriental Medicine, Kyung Hee University, 1 Hoegi-dong, Dongdaemun-gu, Seoul 130-701, Republic of Korea; 3 Department of Otolaryngology, College of Medicine, Kyung Hee University, 1 Hoegi-dong, Dongdaemun-gu, Seoul, 130-701, Republic of Korea; 4 High-Enthalpy Plasma Research Center, Chonbuk National University, 664-14, 1Ga Deokjin-dong, Deokjin-gu, Jeonju, 561-756, Republic of Korea

## Abstract

**Introduction:**

Interleukin (IL)-32 is an inflammatory cytokine induced by *Mycobacterium tuberculosis *and *Mycobacterium bovis *in a variety of cell types and discovered in the synovial of patients with rheumatoid arthritis (RA). Thymic stromal lymphopoietin (TSLP) play several roles in the pathogenesis of RA. However, the role of IL-32 and TSLP in RA has not been elucidated.

**Methods:**

We evaluated the specific mechanism of between IL-32 and TSLP in RA using human monocyte cell line, THP-1 cells.

**Results:**

Here we documented for the first time that IL-32 highly increased TSLP production in THP-1 cells and human blood monocytes. TSLP expression was induced by IL-32 via activation of caspase-1 and nuclear factor-κB. TSLP produced by IL-32 increased differentiation of monocytes but depletion of TSLP prevented differentiation of monocytes into macrophage-like cells. Chondroprotective drugs such as chondroitin sulfate (CS) and the traditional Korean medicine, BaekJeol-Tang (BT) decrease production of TSLP and activation of caspase-1 and nuclear factor-κB. In addition, CS and BT inhibited IL-32-induced monocytes differentiation.

**Conclusions:**

Taken together, IL-32 and TSLP are important cytokines involved in the development of RA. The effects of CS and BT were associated with the downregulation of TSLP and caspase-1 through negative regulation of IL-32 pathways in RA.

## Introduction

Rheumatoid arthritis (RA) is characterized by a chronic inflammation of synovial joints that leads to a progressive destruction of articular and periarticular structures, causing severe morbidity and disability [1]. In RA, the extensive infiltration of inflammatory cells into the synovium and the tumor-like proliferation of RA synovial fibroblasts (RASF) cause the formation of a hyperplastic pannus, which aggressively invades and destroys underlying cartilage and bone. Until now, the role of macrophages, T and B cells, neutrophils, and RASF in the pathophysiology of RA have been examined extensively [2-6].

Interleukin (IL)-32 is a recently described cytokine produced by T lymphocytes, natural killer cells, epithelial cells, mast cells, keratinocytes, eosinophils, and blood monocytes [7-11] and was discovered in the synovial of patients with rheumatoid arthritis but not osteoarthritis [12]. IL-32 was induced by *Mycobacterium tuberculosis *and *Mycobacterium bovis *bacillus Calmette-Guérin as well as by either lipopolysaccharide (LPS) or mycobacteria [13]. Moreover, IL-32 production induced by *M. tuberculosis *is dependent on endogenous interferon-γ (IFN-γ) [13]. A recent study demonstrated that overexpression of the inflammatory mediator, cyclooxygenase 2 resulted in increased IL-32 levels [14]. IL-32 induces the production of tumor necrosis factor (TNF)-α, IL-1β, IL-6, and IL-8 by means of the activation of nuclear factor (NF)-κB, p38 mitogen-activated protein kinase (MAPK), and caspase-1 [7,8,11]. IL-32 also modulates the signals induced by specific Toll-like receptors (TLRs) and nucleotide oligomerization domain (NOD) ligands. IL-32 synergized with NOD1 and NOD2 ligands for the synthesis of IL-1β and IL-6 via activation of caspase-1 [8].

Thymic stromal lymphopoietin (TSLP) is associated with RA, allergic rhinitis and atopic dermatitis [15-17]. It might initiate T helper cell type 2 (Th2) polarization through an OX40-dependent mechanism that affects dendritic cells (DC) activity [17,18]. Signaling of cells by TSLP requires IL-7 receptor [19]. and a distinctive receptor subunit, the TSLP receptor (TSLPR), which is expressed by myeloid DC, monocytes, preactivated T cells, natural killer cells, and mast cells [18,20-22]. In humans, TSLP potently stimulates myeloid DC, with upregulated expression of CD40, CD80, CD86, OX40L, and CD83 and production of chemokines, including thymus and activation-regulated chemokine and macrophage-derived chemokine [23,24]. TSLP was expressed by TNF-α and IL-1β in human airway smooth muscle cells via MAPK, p38 and extracellular signal-regulated kinase (ERK) signaling pathway [25]. Recently, we reported that TSLP was expressed and produced by caspase-1 and NF-κB activation in mast cells [26].

Although the above-mentioned findings indicate a likely role of IL-32 and TSLP in RA, the specific mechanism of between IL-32 and TSLP have not previously been studied. In this study, we report that IL-32 induces TSLP production and monocyte-to-macrophage differentiation through caspase-1. We also investigated the effect of useful therapeutic agents in RA, chondroitin sulfate (CS) and the traditional Korean medicine, BaekJeol-Tang (BT), in IL-32-induced TSLP production and monocyte differentiation.

## Materials and methods

### Reagents

We purchased 3-(4,5-dimethylthiazol-2-yl)-2,5-diphenyltetrazolium bromide (MTT), bicinchoninic acid, caspase-1 inhibitor (CI), pyrrolidine dithiocarbamate (PDTC), lipopolysaccharide (LPS), dimethyl sulfoxide (DMSO), CS, and caspase-1 inhibitor from Sigma-Aldrich (St. Louis, MO, USA); recombinant IL-32, recombinant TSLP, caspase-1 assay kit, and recombinant caspase-1 from R&D Systems (Minneapolis, MN, USA); NF-κB, actin, histone, and caspase-1 antibodies from Santa Cruz Biotechnology (Santa Cruz, CA, USA); CD11b and CD14 antibodies from eBioscience (San Diego, CA, USA); TSLP SMART pool from Dharmacon Inc. (Chicago, IL, USA).

### Preparation of BT and CS

BT is composed of shark cartilage (24 g), Atractylodis Rhizoma (*Atractylodes lancea *De Candolle, 8 g), Phellodendri Cortex (*Phellodendron amurense *Ruprecht, 8 g), and Sophora Radix (*Sophora flavescens Solander *ex Aiton, 8 g). BT (voucher number 201101) was obtained from TeunTeunMaDi Korean Medical Clinic (Seoul, Republic of Korea) and identified by Dr Hyung-Min Kim of the College of Oriental Medicine, Kyung Hee University. An extract of BT was prepared by decocting the dried prescription of herbs with boiling distilled water (48 g/l). The product was filtered, lyophilized and kept at 4°C. The yield of dried product from starting materials was about 35.6%. CS is a major component of shark cartilage. The samples were dissolved in distilled water and then filtered through a 0.22 μm syringe filter.

### Cells

Human monocyte cell line, THP-1 was obtained from the American Type Culture Collection (TIB-202; Manassas, VA, USA). THP-1 cells were cultured in RPMI 1640 supplemented with 10% fetal bovine serum (FBS) and 2 mM glutamine and were maintained at a concentration between 2 and 10 × 10^5 ^cells per milliliter.

### Isolation of peripheral blood mononuclear cells (PBMCs)

PBMCs were isolated from healthy human volunteers. The donors were free of prescribed and over-the-counter medications. After informed consent, PBMCs from heparinized venous blood were isolated with Ficoll gradient centrifugation according to the manufacturer's specification (Sigma-Aldrich, MO, USA). This study was approved by the local ethics committee of Kyung Hee University Hospital in Seoul, Republic of Korea (KMC IRB 1222-06)

### TSLP assay

THP-1 cells were treated with various concentrations of IL-32 for 24 h. The production of TSLP was measured from cells culture supernatant according to the manufacturer's specifications (R&D Systems, Minneapolis, MN, USA).

### Reverse transcription PCR analysis

Using an easy-BLUE™ RNA extraction kit (iNtRON Biotechnology, Republic of Korea), we isolated the total RNA from THP-1 cells in accordance with the manufacturer's specification. The concentrations of total RNA in the final elutes were determined by spectrophotometer. Total RNA (1 mg) was heated at 65°C for 10 min and then chilled on ice. Each sample was reverse-transcribed to cDNA for 90 min at 37°C using a cDNA synthesis kit (Amersham Pharmacia Biotech, Piscataway, NJ, USA). The PCR was performed with the following primer for human TSLP (5' TAT GAG TGG GAC CAA AAG TAC CG 3'; 5' GGG ATT GAA GGT TAG GCT CTG G 3') and GAPDH (5'CAA AAG GGT CAT CAT CTC TG 3'; 5'CCT GCT TCA CCA CCT TCT TG 3'). The annealing temperature was 62°C for TSLP and GAPDH. Products were electrophoresed on a 2% agarose gel and visualized by staining with ethidium bromide.

### Quantitative real-time PCR analysis

Quantitative real-time PCR was performed using a SYBR Green Master Mix and the detection of mRNA was analyzed using an ABI StepOne Real-Time PCR System (Applied Biosystems, Foster City, CA, USA). Primer sequences for the reference gene GAPDH and the genes of interest were as follows: GAPDH (5'TCG ACA GTC AGC CGC ATC TTC TTT 3'; 5'ACC AAA TCC GTT GAC TCC GAC CTT 3'); human TSLP (5 CCC AGG CTA TTC GGA AAC TCA G 3'; 5' CGC CAC AAT CCT TGT AAT TGT G 3'); human CD11b (5'ACG TAA ATG GGA CAA GCT G 3'; 5'GAT CCT GAG GTT CCG TGA AA 3'); human CD14 (5'ACT TGC ACT TTC CAG CTT GC 3'; 5'GCC CAG TCC AGG ATT GTC AG 3'). Typical profile times used were the initial step, 95°C for 10 min followed by a second step at 95°C for 15 s and 60°C for 30 s for 40 cycles with a melting curve analysis. The level of target mRNA was normalized to the level of the GAPDH and compared with the control. Data were analyzed using the ΔΔCT method.

### Caspase-1 enzymatic activity assay

Caspase-1 enzymatic activity was measured according to the manufacturer's specification by using a caspase assay kit.

### Western blot analysis

The stimulated cells were lysed and separated through 10% SDS-PAGE. After electrophoresis, the protein was transferred to nitrocellulose membranes and then the membranes were blocked and incubated with primary and secondary antibodies. Finally, the protein bands were visualized by an enhanced chemiluminesence assay purchased from Amersham Co. (Newark, NJ, USA) following the manufacturer's instructions.

### Transient transfection and luciferase assay

For the transfection, we seeded THP-1 cells (1 × 10^7^) in a 100 mm culture dish. We then used Lipofectamine™ 2000 purchased from Invitrogen (Carlsbad, CA, USA) to transiently transfect pNF-κB luciferase (LUC) and pSV40-LUC reporter gene constructs into THP-1 cells. To measure the luciferase activity, we used a luminometer 1420 luminescence counter purchased from Perkin Elmer (Waltham, MA, USA) in accordance with the manufacturer's protocol. All the transfection experiments were performed in at least three different experiments, with similar results. The relative luciferase activity was defined as the ratio of *firefly *luciferase activity to *renilla *luciferase activity.

### Analysis of monocyte surface antigens by flow cytometry and confocal laser scanning microscopy

THP-1 cultured in the presence or absence of IL-32, TSLP, CI, CS, and BT for 6 days were washed in fluorescence-activated cell sorter (FACS) buffer (phosphate-buffered saline supplemented with 1% bovine serum albumin and 0.1% NaN) and then incubated with 2 μl of fluorescein isothiocyanate (FITC)-conjugated CD14 and phycoerythrin (PE)-conjugated CD11b antibodies for 30 min at 4°C. After washing with FACS buffer, cells were fixed with 1% (weight/volume) paraformaldehyde for 30 min and then stored in the dark until analyzed by flow cytometry. Cytofluorometry was performed with a FACScan (Becton Dickinson, Mountain View, CA, USA). All specimens were examined with a confocal laser scanning microscope.

### Statistical analysis

All results are expressed as the mean ± SEM. The statistical evaluation of the results was performed by an independent *t *test and an ANOVA with a Tukey post hoc test. The results are significant with a value of *P *< 0.05.

## Results

### Upregulation of TSLP production and mRNA expression in IL-32-treated THP-1 cells

**Figure 1:**
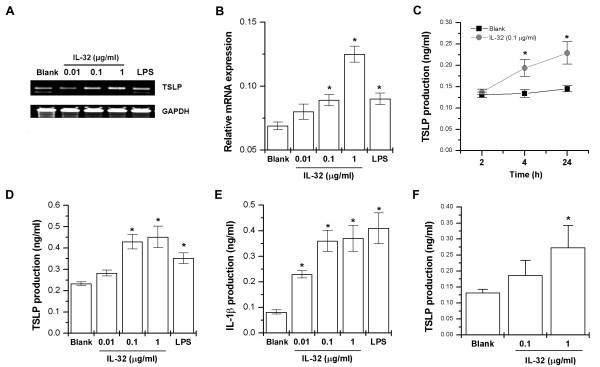
**IL-32-induced mRNA expressions and protein production of TSLP in THP-1 cells**. THP-1 cells (3 × 10^6^) were treated with IL-32 (0.01, 0.1, and 1 μg/ml) or LPS (10 ng/ml) for 5 h. The TSLP mRNA expression was measured by the RT-PCR **(A) **and real-time PCR **(B) **method. **(C) **THP-1 cells (1 × 10^5^) were stimulated with IL-32 (0.1 μg/ml) for various times. **(D) **THP-1 cells (3 × 10^5^) were stimulated with IL-32 (0.01, 0.1, and 1 μg/ml) or LPS for 24 h. The production of TSLP in the supernatant was measured by the ELISA method. **(E) **The production of IL-1β in the supernatant was measured by the ELISA method. **(F) **PBMCs (3 × 10^5^) were stimulated with IL-32 (0.1, and 1 μg/ml) for 24 h. The production of TSLP in the supernatant was measured by the ELISA method. Each datum represents the mean ± SEM of three independent experiments. Blank, unstimulated cells. *P < 0.05; significantly different from unstimulated cells' value. ELISA, enzyme-linked immunosorbent assay; IL, interleukin; LPS, lipopolysaccharide; PBMC, peripheral blood mononuclear cells; RT-PCR, reverse transcriptase polymerase chain reaction; TSLP, thymic stromal lymphopoietin.

IL-32 and TSLP levels are increased in patients with RA [12,15]. To identify the relationship between IL-32 and TSLP in RA, we investigated whether the mRNA of TSLP were expressed by IL-32 in THP-1 cells. When we stimulated the THP-1 cells with IL-32 of various concentrations, the TSLP mRNA was expressed (Figure 1A and 1B). LPS also induced expression of TSLP mRNA. IL-32 significantly increased TSLP production in a time-dependent manner (Figure 1C). TSLP production was significantly increased by IL-32 (0.1 and 1 μg/ml) in a dose-dependent manner (Figure 1D, *P *< 0.05). In our previous work, we reported that IL-32 produced IL-1β. IL-1β production was assayed to test the activity of IL-32. IL-1β production was significantly increased by IL-32 (Figure 1E, *P *< 0.05). IL-32 (1 μg/ml) also significantly increased TSLP production in PBMCs (Figure 1F, *P *< 0.05).

### Caspase-1 and NF-κB involvement in IL-32-induced TSLP production and mRNA expression

**Figure 2:**
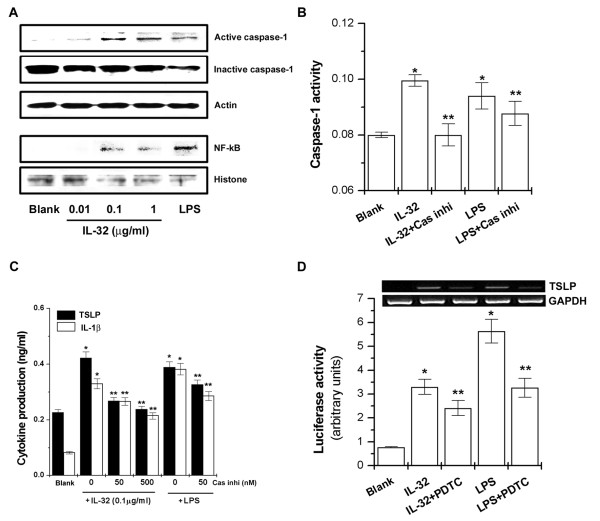
**IL-32-induced TSLP production and mRNA expression via activation of caspase-1 and NF-κB**. **(A) **Cells (3 × 10^6^) were treated with IL-32 (0.01, 0.1, and 1 μg/ml) or LPS (10 ng/ml) for 2 h. Caspase-1 and nuclear NF-κB were determined by Western blot analysis. **(B) **Cells (3 × 10^6^) were treated with caspase-1 inhibitor (50 nM) for 1 h and then stimulated with IL-32 (0.1 μg/ml) or LPS (10 ng/ml) for 2 h. Caspase-1 activity was determined by a colorimetric kit. **(C) **Cells (3 × 10^5^) were treated with caspase-1 inhibitor (50 and 500 nM) for 1 h and then stimulated with IL-32 or LPS for 24 h. TSLP and IL-1β production was determined by ELISA method. **(D) **Cells were treated with PDTC (10 μM) for 1 h and then stimulated with IL-32 or LPS for 5 h. The mRNA expressions TSLP were measured by the RT-PCR method (upper). The NF-κB-luciferase activity was assayed by luciferase assay (lower). Blank, unstimulated cells; Cas inhi, caspase-1 inhibitor 50 nM. *P < 0.05; significantly different from the unstimulated cells' value, **P < 0.05; significantly different from the IL-32 or LPS-stimulated cells' value. ELISA, enzyme-linked immunosorbent assay; IL, interleukin; LPS, lipopolysaccharide; NF-κB, nuclear factor-κB; PBMC, peripheral blood mononuclear cells; PDTC, pyrrolidine dithiocarbamate; RT-PCR, reverse transcriptase polymerase chain reaction; TSLP, thymic stromal lymphopoietin.

Recently, we reported that TSLP was produced and expressed by caspase-1 and NF-κB activation in mast cells [26]. To evaluate whether IL-32 increased caspase-1 and NF-κB activity in THP-1 cells, we performed Western blot analysis and caspase-1 assay. As shown in Figure 2A, IL-32 increased caspase-1 and NF-κB activation. Caspase-1 activity increased by IL-32 was inhibited by caspase-1 inhibitor (Figure 2B). TSLP and IL-1β production increased by IL-32 (0.1 μg/ml) was dose-dependently inhibited by treatment of caspase-1 inhibitor (Figure 2C, *P *< 0.05). NF-κB inhibitor, PDTC, inhibited IL-32-induced NF-κB activation and TSLP mRNA expression (Figure 2D). LPS also induced TSLP production through caspase-1 and NF-κB activation in THP-1 cells.

### Role of caspase-1 in IL-32-induced macrophage-like cells differentiation

**Figure 3:**
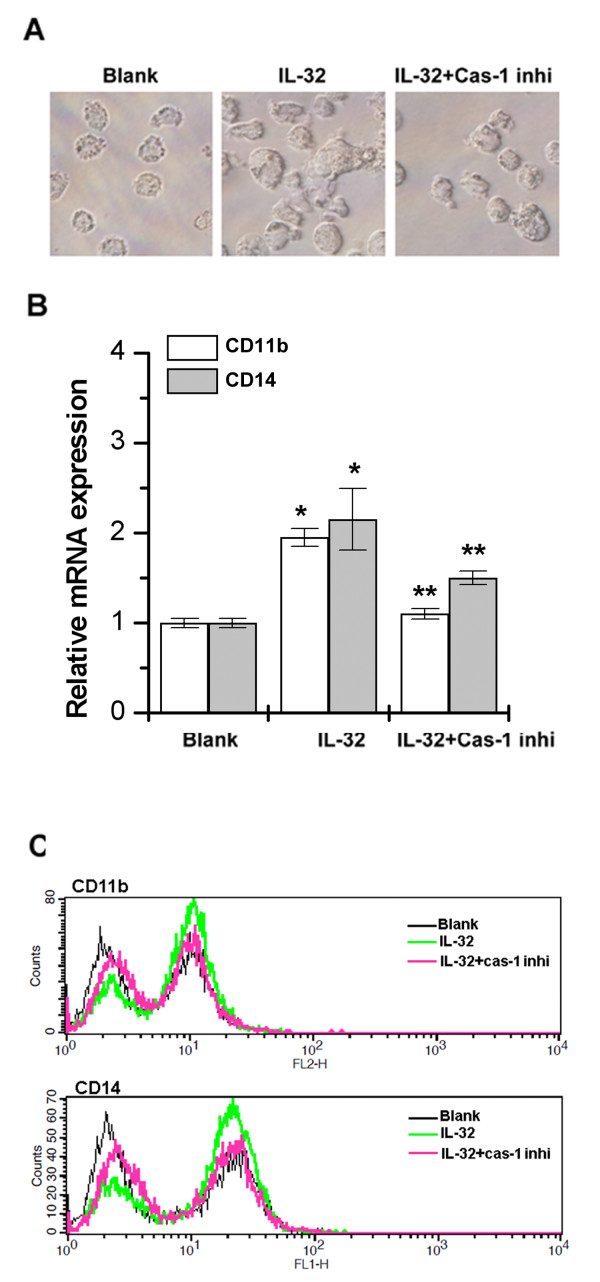
**IL-32-induced THP-1 differentiation via activation of caspase-1**. THP-1 cells were treated with recombinant IL-32 (0.1 μg/ml) or caspase-1 inhibitor (50 nM) for 6 days in the absence of FBS. **(A) **Individual THP-1 cells were photographed. **(B) **Real-time PCR of macrophage markers, CD11b and CD14 after simulation of THP-1 cells. **(C) **FACS analysis of protein expression of macrophage markers, CD11b and CD14. Blank, unstimulated cells; Cas-1 inhi, caspase-1 inhibitor. *P < 0.05; significantly different from the unstimulated cells' value, **P < 0.05; significantly different from the IL-32-stimulated cells' value. (Original magnification × 400). FACS, fluorescence-activated cell sorter; FBS, fetal bovine serum; IL, interleukin; PCR, polymerase chain reaction.

Netea *et al*. reported that IL-32 induces differentiation of monocytes into macrophage-like cells [27]. As shown in Figure 3A, IL-32-treated cells appeared differentiated into macrophages. In contrast, caspase-1 inhibitor blocked IL-32-induced differentiation (Figure 3A). Next, we performed detailed analyses of macrophage markers. As shown in Figure 3B, IL-32 induced transcription of both CD11b and CD14 mRNA but caspase-1 inhibitor significantly reduced CD11b and CD14 mRNA levels in THP-1 cells (Figure 3B). To observe the consistency of the IL-32 effect on surface markers of the macrophage phenotype, THP-1 cells were incubated with IL-32 for 6 days, and the protein expression of CD11b and CD14 was analyzed by FACS. As shown in Figure 3C, IL-32 induced a consistent increase in the protein expression of both CD11b and CD14 compared with control cells incubated with medium only. In contrast, caspase-1 inhibitor reduced CD11b and CD14 protein expression (Figure 3C). When THP-1 cells were cultured for 6 days without FBS, cell death was observed in PDTC-treated THP-1 cells (data not shown).

### Role of TSLP in IL-32-induced macrophage-like cells differentiation

**Figure 4:**
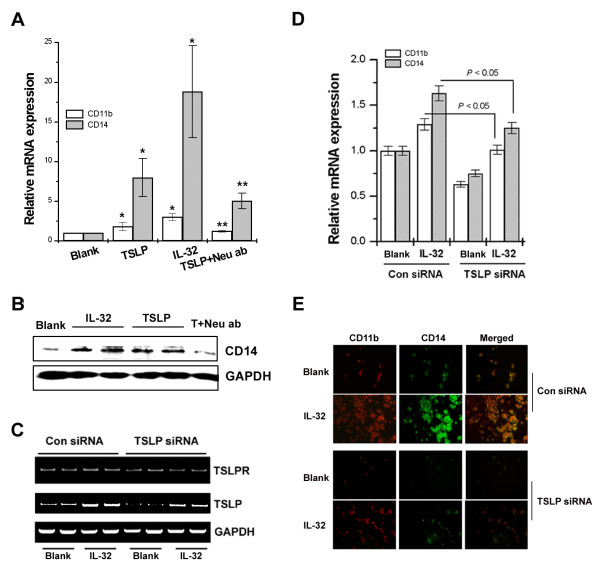
**TSLP increased the expression of CD11b and CD14**. THP-1 cells (3 × 10^5^) were treated with IL-32 (0.1 μg/ml), recombinant TSLP (20 ng/ml), or TSLP neutralizing antibodies (1 μg/ml) for 6 days in RPMI1640 containing 10% FBS. **(A) **Total RNA was assayed by real-time PCR for CD11b and CD14. **(B) **CD14 was determined by Western blot analysis. THP-1 cells were transfected with TSLP siRNA. Transfected THP-1 cells (3 × 10^5^) were treated with IL-32 (0.1 μg/ml) or recombinant TSLP (20 ng/ml) for 6 days. **(C) **The total RNA was assayed by RT-PCR analysis for TSLPR and TSLP mRNA. **(D) **Real-time PCR of macrophage markers, CD11b and CD14 after simulation of THP-1 cells. **(E) **CD11b (red) and CD14 (green) were examined with a confocal laser scanning microscope. Blank, unstimulated cells; Neu ab, TSLP neutralizing antibodies. *P < 0.05; significantly different from the unstimulated cells' value, **P < 0.05; significantly different from the TSLP-stimulated cells' value. (Original magnification × 600). FBS, fetal bovine serum; IL, interleukin; RT-PCR, reverse transcriptase polymerase chain reaction; TSLP(R), thymic stromal lymphopoietin (receptor).

To investigate the direct role of TSLP in IL-32-induced macrophage-like cells differentiation, THP-1 cells were stimulated with recombinant TSLP. As shown in Figure 4A, recombinant TSLP significantly increased CD11b and CD14 mRNA expression but increased CD11b and CD14 mRNA expression were reduced by neutralizing anti-TSLP antibodies. The expression of CD14 mRNA by TSLP is higher than that of CD11b. Therefore, we examined the level of CD14 protein expression by TSLP. TSLP also increased expression of CD14 protein (Figure 4B). TSLP signals via TSLP receptor (TSLPR), a heterodimer of the IL-7 receptor alpha chain (IL-7Rα) and the TSLPR chain supports differentiation and survival of various immune cells such as DC, T cells, and B cells [28]. TSLPR, which is expressed by myeloid DC, monocytes, preactivated T cells, natural killer cells, and mast cells [19]. As shown in Figure 4C, TSLPR was expressed in THP-1 cells. In order to confirm whether differentiation of THP-1 cells was mediated by TSLP, we depleted TSLP in THP-1 cells using TSLP siRNA (TSLP SMARTpool, Dharmacon Inc., Chicago, IL, USA). Messenger RNA analysis by RT-PCR revealed that TSLP was reduced (Figure 4C). The expressions of CD11b and CD14 were also decreased by TSLP siRNA (Figure 4D and 4E). Confocal laser scanning microscopic analysis clearly demonstrated that the enhanced CD11b and CD14 protein expressions were induced by treatment with IL-32, but it was markedly blocked by TSLP siRNA (Figure 4E).

### Effect of BT and CS on IL-32 or LPS-induced TSLP production and mRNA expression

**Figure 5:**
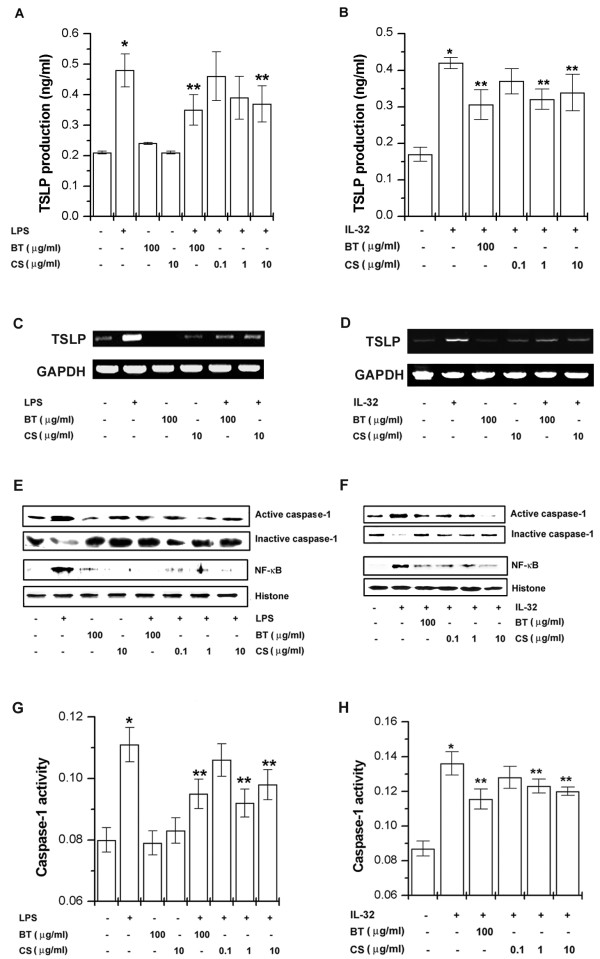
**BT and CS inhibited the IL-32 or LPS-induced TSLP production and mRNA expression through the inhibition of caspase-1 and NF-κB activation**. THP-1 cells (3 × 10^5^) were treated with BT (100 μg/ml) and CS (0.1, 1, and 10 μg/ml) for 2 h and then stimulated with LPS (10 ng/ml, **(A) **or IL-32 (0.1 μg/ml, **(B) **for 24 h. The production of TSLP in the supernatant was measured by the ELISA method. **(C **and **D) **THP-1 cells (3 × 10^6^) were treated with BT or CS for 5 h. The mRNA expressions of TSLP were measured by the RT-PCR method. THP-1 cells (3 × 10^6^) were treated with BT (100 μg/ml) and CS (0.1, 1, and 10 μg/ml) for 2 h and then stimulated with IL-32 (0.1 μg/ml) or LPS (10 ng/ml) for 2 h. **(E **and **F) **Caspase-1 and NF-κB were determined by Western blot analysis. **(G **and **H) **Caspase-1 activity was determined by a colorimetric kit. *P < 0.05; significantly different from the unstimulated cells' value, **P < 0.05; significantly different from the IL-32 or LPS-stimulated cells' value. BT, BaekJeol-Tang; CS, chondroitin sulfate; ELISA, enzyme-linked immunosorbent assay; IL, interleukin; LPS, lipopolysaccharide; NF-κB, nuclear factor-κB; RT-PCR, reverse transcriptase polymerase chain reaction; TSLP, thymic stromal lymphopoietin.

Chondroprotective drugs such as BT and CS is used for the treatment of arthritis such as RA and osteoarthritis. We assessed the regulatory effect of BT and CS on IL-32 or LPS-induced TSLP production and mRNA expression. The results showed that BT and CS significantly inhibited IL-32 or LPS-induced TSLP production and mRNA expression (Figures 5A to 5D, *P *< 0.05). CS dose-dependently inhibited LPS-induced TSLP production. BT or CS did not affect TSLP production by itself.

### Effect of BT and CS in IL-32 or LPS-induced caspase-1 and NF-κB activation

We investigated whether BT and CS would influence the activation of caspase-1 induced by IL-32 or LPS in THP-1 cells. Western blot analysis for caspase-1 was performed. Caspase-1 activation by IL-32 or LPS was decreased by the treatment of BT or CS (Figure 5E and 5F). We also estimated the effect of BT and CS on caspase-1 activation using a caspase-1 assay kit. Once again, this showed that caspase-1 activity was significantly inhibited by the treatment of BT or CS (Figure 5G and 5H, *P *< 0.05). The expression of TSLP mRNA was regulated by the transcription factor, NF-κB [26]. Because the suppression of NF-κB is linked with anti-inflammation, we postulated that BT and CS mediate its effects at least partly through the suppression of NF-κB activation. To assess the regulatory mechanism of BT and CS on TSLP mRNA expression, we examined the regulatory effect of BT and CS on the translocation of NF-κB into the nucleus. The stimulation with IL-32 or LPS increased translocated levels of NF-κB in the nucleus but the translocated levels of NF-κB were suppressed by the treatment of BT or CS (Figure 5E and 5F).

### Effect of BT and CS in IL-32-induced macrophage-like cells differentiation

**Figure 6:**
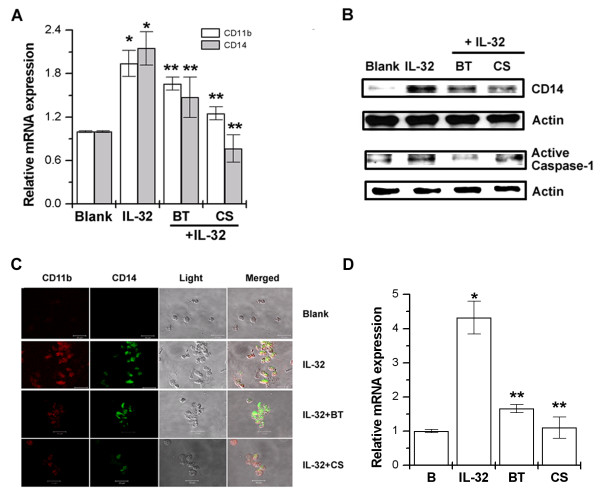
**BT and CS inhibited the IL-32-induced THP-1 differentiation**. THP-1 cells (3 × 10^5^) were treated with BT (100 μg/ml) or CS (1 μg/ml) for 2 h and then stimulated with IL-32 (0.1 μg/ml) for 6 days in the absence of FBS. **(A) **Real-time PCR of macrophage markers, CD11b and CD14 mRNA after simulation of THP-1 cells. **(B) **CD14 and caspase-1 were determined by Western blot analysis. **(C) **CD11b (red) and CD14 (green) were examined with confocal laser-scanning microscope. **(D) **The mRNA expressions of TSLP were measured by the real-time PCR. Blank, unstimulated cells. *P < 0.05; significantly different from the unstimulated cells' value, **P < 0.05; significantly different from the IL-32-stimulated cells' value. (Original magnification × 600). BT, BaekJeol-Tang; CS, chondroitin sulfate; FBS, fetal bovine serum; PCR, polymerase chain reaction; TSLP, thymic stromal lymphopoietin.

Finally, we assessed the regulatory effect of BT and CS in IL-32-induced macrophage-like cells differentiation. The expression of CD11b and CD14 mRNA was analyzed by real-time PCR. BT and CS inhibited expressions of CD11b and CD14 mRNA and protein (Figure 6A and 6B). Confocal laser scanning microscopic analysis clearly demonstrated that the enhanced expression of CD11b and CD14 was induced by the treatment of IL-32, but it was markedly blocked by the treatment of BT or CS (Figure 6C). BT and CS significantly inhibited IL-32-induced TSLP mRNA expression (Figures 6D, *P *< 0.05).

## Discussion

In the present study, we demonstrated that IL-32 induced the TSLP production and mRNA expression via activation of caspase-1 and NF-κB. IL-32 and TSLP also increased the differentiation of THP-1 cells. In addition, CS and BT inhibited the differentiation of THP-1 cells through inhibition of TSLP production and caspase-1 activation.

IL-32 plays an important role in host defense against microorganisms including Mycobacterium, HIV, and influenza [13,29-31]. IL-32 has also been implicated in several inflammatory disorders, including rheumatoid arthritis, Crohn's disease, atopic dermatitis, chronic obstructive pulmonary disease, and allergic rhinitis [9,11,12,32,33]. IL-32 expression is related with disease severity in these conditions. Recently, we have been shown that silencing of endogenous IL-32 results in the reduction of IL-6, IL-8, TNF-α, and vascular endothelial growth factor (VEGF) [11]. TSLP produced the IL-5, IL-6, IL-13, and granulocyte macrophage colony-stimulating factor from mast cells [18]. TSLP expression correlates with markers of inflammation. TSLP-induced immune activation increased the joint destruction, because TSLPR-/-mice displayed decreased cartilage and bone erosion, decreased number of tartrate-resistant acid phophatase-positive osteoclasts, and reduced local expression of factors that are involved in tissue destruction [15]. Block of TSLP reduced the severity of collagen-induced arthritis [15]. From this, we can presuppose that TSLP produced by IL-32 plays an important role in expression of inflammatory cytokines. These properties suggest that IL-32-induced TSLP might play an important role in the amplification of inflammatory reactions such as RA. Future studies will be required to investigate whether TSLP acts as an endogenous regulator of proinflammatory cytokine production in monocytes.

It has been shown that IL-32 potentiates one or more of the intracellular pathways induced by NOD ligands. Synthetic muramyl dipeptide engagement of NOD2 results in the activation of two signaling pathways. First, NOD2 activates the serine/threonine kinase receptor-interacting protein 2/RICK/CARDIAK, and this leads to NF-κB activation and transcription of proinflammatory cytokine genes such as those encoding TNF-α, IL-1β, and IL-6 [34]. Second, the CARD domain of NOD-like receptors proteins interacts with caspase-1 and 5, and this interaction results in the processing of cytokine precursors such as pro-IL-1β and pro-IL-18 [34]. In this study, IL-32 increased the caspase-1 and NF-κB activation in THP-1 cells. Caspase-1 inhibitor or NF-κB inhibitor reduced the IL-32-induced TSLP production and mRNA expression. Taken together, these reports imply that IL-32 induces TSLP production and mRNA expression in THP-1 cells through a signaling cascade downstream of the caspase-1 and NF-κB activation.

Both the innate and acquired immune systems rely heavily upon the effector and regulatory activities of monocytes and macrophages [35]. Differentiation of myeloid cells involves broad-based and tightly regulated changes in gene expression induced in response to diverse cytokines, growth factors and other agonists [36,37]. Expression of CD11b and CD14 is upregulated during myeloid cell differentiation. Myeloid zinc finger-1 is involved downstream of phosphatidylinositide 3-kinase in a calcitriol-induced signaling pathway leading to myeloid cell differentiation and activation of CD11b and CD14 [37]. Recent studies by Netea and colleagues reported that IL-32 induced a consistent increase in the expression of both CD1a and CD14 [27]. TSLP is capable of activating dendritic cells to promote Th2 immune responses. TSLP has also been shown to directly promote Th2 differentiation of naïve CD4 (+) T cell and activate natural killer T cells, basophils and other innate immune cells at the initial stage of inflammation [38]. We also observed that IL-32 induces differentiation of THP-1 cells into macrophage-like cell. Netea *et al*., showed caspase-3 activation, but not caspae-1, -8, -9, plays an important role in monocyte differentiation [27]. However, we investigated that caspase-1 inhibitor reduced the CD11b and CD14 expression through the inhibition of TSLP production. We also showed that TSLP siRNA inhibited the monocyte differentiation. For that reason, we suggest that blockade of IL-32 and TSLP signaling may be effective in prevention and treatment in models of inflammatory arthritis.

The suppressing of TSLP production signaling in THP-1 cells may be a useful tool to reduce monocyte differentiation in inflammatory conditions. CS belongs to the class of natural complex polysaccharides and is an unbranched, polydisperse, complex macromolecule extracted and purified from various tissues [39]. It is a useful therapeutic agent in diseases such as inflammatory bowel diseases, artherosclerosis, Parkinson's and Alzheimer's diseases, multiple sclerosis, amyotrophic lateral sclerosis, rheumatoid arthritis, and systemic lupus erythematosus [40]. CS diminishes the expression of inflammatory proteins via inhibiting of NF-κB nuclear translocation in chondrocyte, synovial macrophages, synoviocytes, and peripheral blood mononuclear cells [40]. In previous studies, David-Raoudi *et al*., reported that CS increases hyaluronan production through upregulation of the expression of hyaluronan synthases [39]. BT, a traditional Korean medicine, has long been prescribed as a treatment for joint diseases to protect injured chondrocytes. However, effect of these drugs has not been elucidated on TSLP production and monocyte differentiation. In this study, we showed that BT or CS significantly inhibited the production of TSLP, activation of caspase-1 and NF-κB, and differentiation of monocytes in THP-1 cells. Therefore, our finding provides new evidence that chondroprotective effect caused by the treatment of BT and CS is related to the inhibition of TSLP production and monocyte differentiation.

## Conclusions

In conclusion, we show for the first time that IL-32 induces TSLP production by increasing NF-κB and caspase-1 activation. IL-32-induced TSLP induces differentiation of monocytes into macrophage-like cells with characteristic surface markers (CD11b and CD14). To our knowledge, this is also the first study to demonstrate that chondroprotective effect of BT and CS is accompanied by a regulation of TSLP production. Our results have demonstrated an essential role for the IL-32/TSLP pathway in RA. Further investigation is necessary to determine other possible mechanisms of IL-32 and TSLP and to apply it clinically in RA and other inflammatory diseases.

## Abbreviations

BT: BaekJeol-Tang; CI: caspase-1 inhibitor; CS: chondroitin sulfate; DC: dendritic cells; DMSO: dimethyl sulfoxide; ERK: extracellular signal-regulated kinase; FACS: fluorescence-activated cell sorter; FBS: fetal bovine serum; IFN-γ: interferon-γ; IL-32: interleukin-32; LPS: lipopolysaccharide; MAPK: mitogen-activated protein kinase; MTT: 3-(4,5-dimethylthiazol-2-yl)-2,5-diphenyltetrazolium bromide; NF-κB: nuclear factor-κB; NOD: nucleotide oligomerization domain; PBMC: peripheral blood mononuclear cells; PDTC: pyrrolidine dithiocarbamate; RA: rheumatoid arthritis; RT-PCR: reverse transcriptase time polymerase chain reaction; siRNA: small interfering RNA; TLR: toll-like receptors; TNF: tumor necrosis factor; TSLP: thymic stromal lymphopoietin; TSLPR: TSLP receptor.

## Competing interests

The authors declare that they have no competing interests.

## Authors' contributions

HJJ and HMK designed and wrote the manuscript. HJJ and SYN did the experiments and analysis. HAO did the analysis of the confocal microscopy. NRH and PDM did the real-time PCR and ELISA. SYS isolated the PBMCs. MHK and YSK provided comments on the manuscript. HMK supervised the research. All authors read and approved the final manuscript.
